# Characteristics of complementary medicine therapists in Switzerland: A cross-sectional study

**DOI:** 10.1371/journal.pone.0224098

**Published:** 2019-10-23

**Authors:** Julie Dubois, Anne-Sylvie Bill, Jérôme Pasquier, Silva Keberle, Bernard Burnand, Pierre-Yves Rodondi

**Affiliations:** 1 Center for Primary Care and Public Health (Unisanté), University of Lausanne, Lausanne, Switzerland; 2 Institute of Family Medicine, University of Fribourg, Fribourg, Switzerland; 3 Empirical Medicine Register, Basel, Switzerland; Charles Sturt University, AUSTRALIA

## Abstract

**Objective:**

More than 27,000 complementary medicine (CM) therapists are registered in Switzerland, but limited data are available on their occupational profile and role in the healthcare system. Herein we aimed to gain a better understanding of the professional profile of non-physician licensed therapists, focusing on acupuncture, osteopathy, and European naturopathy.

**Design:**

This cross-sectional study was based on an online anonymous survey conducted from March to June 2017.

**Setting and participants:**

All 1549 non-physician registered osteopaths, acupuncturists, and naturopaths in the French-speaking part of Switzerland were asked to complete the survey.

**Main outcome measures:**

We measured the therapists’ demographics, training and practice characteristics, and workload.

**Results:**

A total of 426 therapists returned the questionnaire (response rate: 27.5%). The mean age of the respondents was 46.0 years (SD 11.6) and most were women (67.8%). CM represented the main professional activity for a majority of therapists (82.8%), most of whom were independent (86.3%). The length and number of consultations per month varied across professions. Multivariate analysis showed that acupuncturists and naturopaths performed significantly fewer consults per month than osteopaths did. However, consultation length was significantly longer for acupuncturists and naturopaths than for osteopaths. Acupuncturists (71.6%) and naturopaths (64.4%) were significantly more favorable than osteopaths (27.7%) to have consultation costs covered by basic health insurance. Professional profiles differed between osteopaths, on the one hand, and naturopaths and acupuncturists, on the other, mainly regarding workload, treatment duration, and main reasons to consult.

**Conclusions:**

This first study to investigate a variety of therapist profiles in Switzerland provides useful information about their activities and role within the Swiss healthcare system. Although all three professions are encompassed under the same umbrella term (CM), our study showed that they have specific work cultures and areas of intervention in the healthcare system.

## Introduction

In the United States in 2012, 33% of the population aged 18 years or more used some type of complementary medicine (CM) in the previous 12 months [1]. In Australia, the prevalence of use was 63.1% among people aged 18 or more in 2017 [2]. In Europe, the prevalence of CM use is hard to estimate because of the heterogeneity in the quality of studies and in the definitions of CM used [3]. In Switzerland, the Swiss Health Survey showed that, in 2012, 25% of the population aged 15 or more had used some CM approach at least once in the previous 12 months [4]. Basic health insurance there covers the following five CM treatments when provided by a trained physician: acupuncture, traditional Chinese medicine (TCM), anthroposophic medicine, homeopathy, and herbal medicine. Many other CM treatments, including treatments performed by registered non-physician therapists, are covered by private supplemental insurance plans that have various conditions for reimbursement of these treatments [4].

In Europe, a 2012 study estimated that there were at least 300,000 registered CM therapists, less than half of them physicians [5]. That same study reported that there were at least 65 CM therapists, as opposed to 95 family physicians, per 100,000 inhabitants in Europe [5]. In Switzerland, there are currently around 27,000 registered CM therapists in the two main registers that offer accreditations in the domain of CM: the Empirical Medicine Register (EMR) and the Swiss Foundation for Complementary Medicine (ASCA). These accreditations provide recognition to therapists and allow them to be reimbursed by supplemental private health insurance plans. By comparison, there were 36,900 physicians in the country in 2017, of whom 18,858 were ambulatory physicians [6]. This amounts to 438 physicians and 320 CM therapists per 100,000 inhabitants.

Despite the growing body of literature on CM, little attention has been paid to the providers. In the United States, researchers have investigated the characteristics of acupuncturists, massage therapists, chiropractors, and naturopathic physicians [7, 8]. Such studies have provided useful descriptive information about curricula and the sociodemographic and practice profiles of therapists in the various states within the country. The profiles of CM therapists have been investigated more extensively in Australia and New Zealand through surveys conducted among massage therapists, TCM therapists, osteopaths, and naturopaths [9–18]. In Europe, studies have mainly described the characteristics of acupuncturists and osteopaths, but most of those studies focused solely on the type of care delivered and on patient profiles [19–25]. Despite the substantial number of CM therapists and the high prevalence of CM use in the population, few data are available on the occupational profiles of CM therapists, especially in Europe.

With increased attention directed at these professions, there has been growing interest in assessing the current state of the practices of CM therapists, as well as their place within the Swiss health system. Thus, the aim of this study was to gain a better understanding of registered non-physician licensed therapists in the French-speaking part of Switzerland by investigating their professional profiles (including training, practice characteristics and patients’ profile). The study was limited to non-physician CM therapists, as they are less integrated in the conventional medical system. Because numerous different CM therapies coexist in Switzerland and it is impossible to describe the therapists’ profiles in every one of these disciplines, we focused on three popular methods: naturopathy (European), acupuncture, and osteopathy.

## Method

### Study design

We conducted this cross-sectional observational study by using an online anonymous practice-based survey between March and June 2017. The questionnaire was sent to all registered osteopaths, naturopaths, and acupuncturists of the French-speaking part of Switzerland.

### Setting and participants

Most therapists are registered in one of the two largest private organizations that provide accreditations in the domain of CM in Switzerland: EMR or ASCA. Therapists were informed about the research project through an email sent by EMR and ASCA to all acupuncture, European naturopathy, and osteopathy therapist members in the French-speaking part of Switzerland. The email, sent only to therapists who indicated that French is their language of correspondence, contained a letter of information about the research and a link to the online questionnaire. Two reminders, after 2 weeks and after 1 month, were sent through the same channel to encourage participants to answer. Inclusion criteria were that therapists had to speak French and that they had to be practicing in the French-speaking part of Switzerland. The exclusion criterion was being a conventional medicine physician. Two mandatory items in the questionnaire allowed us to check for these criteria.

Some therapists were registered for several therapies (e.g., osteopathy and acupuncture) and thus received the emails twice. As the questionnaire did not allow to check more than one therapy per participant, these participants were asked to refer in their answers to the therapy they practiced the most. Because of these double registrations and the fact that the questionnaire was anonymous, the response rate for each therapy could not be calculated precisely, unlike the general response rate.

### Variables

We developed a 46-item questionnaire divided into nine sections using the Limesurvey software. The first three sections (14 items) explored sociodemographic and training data. The next three sections contained questions on practice characteristics, work environment, and workload (25 items). In the last sections, information such as patients’ main complaints, transmission of information, and treatment coverage were collected (7 items). Participants were asked to indicate the five most common complaints reported by their patients. For the analysis, these complaints were then classified under larger groups (such as pain, sleeping disorders, and digestive disorders). Willingness to have treatments covered by basic health insurance was assessed through a Likert scale. Answers where then grouped (strongly agree, agree, neutral vs. strongly disagree, disagree) to report frequencies.

### Data sources

To ensure that we asked relevant questions about the characteristics of the professions under scope, we conducted one preliminary interview with one practitioner of each of the three professions included in this study. These interviews served as a basis for developing the questionnaire; some items were also adapted from existing questionnaires [10, 19, 21, 26, 27]. The questionnaire was cognitively tested by a sample group of therapists with various sociodemographic characteristics. Amendments were then made to the questionnaire to improve comprehension of the survey items. The therapists participating in the cognitive testing did not take part in the study. The Cantonal Commission for the Ethics of Human Research (CER-VD) approved the study (Reference Req-2016-00535) and waived the need for consent, given the scope and nature of the study and the anonymous data collection and analysis.

### Statistical analysis

Responses to the questionnaire were analyzed with R statistical software (version 3.4.2). Standard descriptive analyses (e.g., means and standard deviations for continuous variables and percentages for categorical variables) were used to summarize sociodemographic variables, training, practice characteristics, and patient profiles. In all of these areas, we conducted two-by-two comparisons among the three professions by using Fisher’s exact tests and the Student’s t-test for categorical and continuous variables, respectively. We conducted bivariate analyses to explore factors associated with therapists’ practices. Associations between continuous variables were estimated with Pearson’s correlation coefficients, whereas associations between binary variables were estimated with the Fisher’s exact test. Associations between binary variables and continuous variables were estimated with the Student’s t-test. We also conducted multivariate analyses to describe associations between therapists’ practices, on the one hand (outcome variables), and profession, age, gender, and interactions between the latter three on the other (explanatory variables). Multivariate linear and logistic regressions were used for numeric and binary outcomes, respectively. Variables and interactions were introduced into the models on the basis of the Akaike information criterion and clinical relevance.

## Results

### Sociodemographic data

Among the 1549 therapists who received the questionnaire, 426 returned it (general response rate: 27.5%; osteopaths: 27.9–28.8%; naturopaths: 32.7–34.9%; acupuncturists: 20.4–21.9%). Most respondents were women (67.8%). The proportion of women was significantly higher among naturopaths than among acupuncturists (p = 0.001) or osteopaths (p<0.001). The mean age of the respondents was 46.0 years (SD 11.6). Osteopaths were significantly younger than acupuncturists (p<0.001) and naturopaths (p<0.001). On average, respondents went into practice around the time of their graduation, as they had graduated 12.6 (SD7.4) years ago and had been practicing on average for 12.7 years (SD 7.6) with no significant difference between the three professions. Detailed sociodemographic data are reported in Table 1.

**Table 1:** Sociodemographic data and training characteristics.

	Overall	Osteopaths	Naturopaths	Acupuncturists
	(N = 426, 100%)	(N = 234, 54.9%)	(N = 104, 24.4%)	(N = 88, 20.7%)
**Sex (n = 422)**				
Male	136 (32.2%)	84 (36.2%)	18 (17.5%)	34 (39.1%)
Female	286 (67.8%)	148 (63.8%)	85 (82.5%)	53 (60.9%)
**Age (n = 409)**, mean (SD)	46.0 (11.6)	41.5 (10.8)	52.6 (10.8)	50.6 (8.9)
**Nationality (n = 423)**				
Switzerland	361 (85.3%)	209 (89.7%)	90 (88.2%)	62 (70.5%)
France	35 (8.3%)	15 (6.4%)	9 (8.8%)	11 (12.5%)
China	6 (1.4%)	0 (0%)	0 (0%)	6 (6.8%)
Italy	6 (1.4%)	3 (1.3%)	2 (2.0%)	1 (1.1%)
Other	15 (3.5%)	6 (2.6%)	1 (1.0%)	8 (9.1%)
**Years since graduation (n = 418)**, mean (SD)	12.6 (7.4)	12.3 (7.0)	12.7 (7.7)	13.2 (7.9)
**Years in practice (n = 423)**, mean (SD)	12.7 (7.6)	12.5 (7.5)	12.7 (7.5)	13.1 (7.9)
**Training country (n = 424)**				
Switzerland	305 (71.9%)	170 (72.6%)	83 (81.4%)	52 (59.1%)
France	75 (17.7%)	49 (20.9%)	12 (11.8%)	14 (15.9%)
China	13 (3.1%)	0	0	13 (14.8%)
England	10 (2.4%)	8 (3.4%)	0	2 (2.3%)
Other	21 (5.0%)	7 (3.0%)	7 (6.9%)	7 (8.0%)
**Additional CM training (n = 389)**				
Yes	129 (33.2%)	22 (9.9%)	63 (72.4%)	44 (55.7%)
No	260 (66.8%)	201 (90.1%)	24 (27.6%)	35 (44.3%)
**Former training (n = 413)**				
No	179 (43.3%)	146 (62.9%)	14 (14.3%)	19 (22.9%)
Yes	234 (56.7%)	86 (37.1%)	84 (85.7%)	64 (77.1%)

CM: complementary medicine.

### Training

Most therapists were trained in Switzerland or France. Only acupuncturists had a slightly different training profile, with 59.1% of them trained in Switzerland and 14.8% trained in China. One third of the participants were also trained in an additional CM modality. Acupuncturists (p<0.001) and naturopaths (p<0.001) were significantly more often trained in another CM modality than osteopaths were (Table 1). Acupuncturists were mainly trained in disciplines pertaining to TCM. Naturopathy was the profession with the highest number of different CM training per therapist (up to seven) (for details on CM training see Table 2).

**Table 2:** Therapists’ other CM training.

Additional CM therapy	Overall (n = 123)	Osteopaths (n = 17)	Naturopaths (n = 63)	Acupuncturists (n = 43)
Massage	28 (22.8%)	3 (17.6%)	22 (34.9%)	3 (7.0%)
Lymphatic drainage	19 (15.4%)	2 (11.8%)	15 (23.8%)	2 (4.7%)
Homeopathy	16 (13.0%)	1 (5.9%)	11 (17.5%)	4 (9.3%)
Reflexology	14 (11.4%)	0 (0%)	13 (20.6%)	1 (2.3%)
TCM	11 (8.9%)	1 (5.9%)	6 (9.5%)	4 (9.3%)
Chinese herbs	10 (8.1%)	0 (0%)	0 (0%)	10 (23.3%)
Tuina	10 (8.1%)	0 (0%)	0 (0%)	10 (23.3%)
Naturopathy	9 (7.3%)	3 (17.6%)	2 (3.2%)	4 (9.3%)
Bioresonance therapy	9 (7.3%)	1 (5.9%)	6 (9.5%)	2 (4.7%)
Aromatherapy	9 (7.3%)	0 (0%)	8 (12.7%)	1 (2.3%)
Nutrition	9 (7.3%)	0 (0%)	8 (12.7%)	1 (2.3%)
Acupuncture	8 (6.5%)	6 (35.3%)	2 (3.2%)	0 (0%)
Phytotherapy	7 (5.7%)	0 (0%)	6 (9.5%)	1 (2.3%)

CM: complementary medicine, TCM: Traditional Chinese medicine

As participants could indicate multiple additional CM training, percentages add up to more than 100%.

When less than 5% of the respondents declared being trained in a specific therapy, the data do not appear in the table.

More than half of the participants had previous training outside the CM field, with naturopaths (p<0.001) and acupuncturists (p<0.001) reporting it significantly more often than osteopaths did. Among those with previous training, osteopaths (67.1%, n = 57) and acupuncturists (57.4%, n = 35) were significantly more likely to be trained in another healthcare-related field than naturopaths (21.7%, n = 18) were (both p<0.001). Among therapists trained in another healthcare-related field, almost two thirds of osteopaths and one third of acupuncturists had training in physiotherapy. Among therapists with previous training outside the CM field, acupuncturists (53.2%; n = 33) were significantly more likely than naturopaths (22.9%; n = 19; p<0.001) and osteopaths (24.4%; n = 20; p<0.001) to still work in that field (for details on training see Table 3).

**Table 3:** Participants’ training outside the CM field.

Training	Overall (n = 229)	Osteopaths (n = 83)	Naturopaths (n = 85)	Acupuncturists (n = 61)
Physiotherapist	69 (30.1%)	52 (61.2%)	0 (0%)	17 (27.9%)
Commercial employee	18 (7.9%)	1 (1.2%)	15 (18.1%)	2 (3.3%)
Secretary	17 (7.4%)	2 (2.4%)	13 (15.7%)	2 (3.3%)
Nurse	15 (6.6%)	1 (1.2%)	6 (7.2%)	8 (13.1%)
Teacher	14 (6.1%)	2 (2.4%)	9 (10.8%)	3 (4.9%)
Midwife	7 (3.1%)	1 (1.2%)	0 (0%)	6 (9.8%)

When less than 3% of the respondents declared a training in a specific profession, the data do not appear in the table.

### Practice characteristics

CM represented the main professional activity for the majority of therapists, especially for osteopaths compared with acupuncturists (p<0.001) and compared with naturopaths (p<0.001). The majority of therapists were self-employed and had one practice location. Overall, therapists worked an average of 30.7 hours per week (SD 12.5), with osteopaths working significantly more hours per week than naturopaths (p<0.001) and acupuncturists (p<0.001). Overall, therapists performed 97.4 monthly consultations (SD 62.7) and the average length of encounters was 55.1 minutes (SD 15.8), but there was a significantly different distribution across professions for both of these aspects (both p<0.001). Detailed results are presented in Table 4.

**Table 4:** Practice characteristics.

	Overall	Osteopaths	Naturopaths	Acupuncturists
	(N = 426, 100%)	(N = 234, 54.9%)	(N = 104, 24.4%)	(N = 88, 20.7%)
**Main professional activity (n = 419)**				
Yes	347 (82.8%)	217 (93.5%)	75 (74.3%)	55 (64.0%)
No or 50/50	72 (17.2%)	15 (6.5%)	26 (25.7%)	31 (36.0%)
**Employment status (n = 422)**				
Self-employed	364 (86.2%)	187 (80.6%)	99 (97.1%)	78 (88.6%)
Employee	42 (10.0%)	36 (15.5%)	1 (1.0%)	5 (5.7%)
Both	16 (3.8%)	9 (3.9%)	2 (2.0%)	5 (5.7%)
**Number of practices (n = 422)**				
1	327 (77.5%)	167 (71.7%)	86 (84.3%)	74 (85.1%)
2	83 (19.6%)	58 (24.9%)	12 (11.8%)	13 (14.9%)
More than two	10 (2.4%)	7 (3.0%)	3 (2.9%)	0
None	2 (0.5%)	1 (0.4%)	1 (1.0%)	0
**Weekly working hours (n = 393)**, mean (SD)	30.7 (12.5)	33.9 (10.1)	27.8 (14.4)	24.6 (13.4)
**Consultations/month (n = 351)**, mean (SD)	97.4 (62.7)	117.5 (51.3)	54.3 (57.1)	83.2 (72.9)
**Consultation length (n = 418)**, mean (SD)	55.1 (15.8)	45.5 (6.5)	72.0 (16.7)	61.6 (12.5)

When asked whether the consultation costs of their therapies should be included in the list of basic health insurance-reimbursed therapies, acupuncturists (71.6%, n = 58)) and naturopaths (64.4%, n = 65) were significantly more in favor than osteopaths were (27.7%, n = 64) (both p<0.001).

### Patient profile

The mean proportion of female patients reported by the therapists (n = 418) was 67.9% (SD 12.0). Osteopaths (n = 231) reported having significantly fewer female patients (63.3%; SD±8.6) than acupuncturists (73.1%; SD 12.8, n = 85) and naturopaths did (74.0%; SD 13.5, n = 102) (both p<0.001). All therapists reported having patients that were mainly aged 19–64 (61.5% of all patients, SD 17.5%) n = 417). Osteopaths (n = 229) reported having the most pediatric patients (aged 0–18 years), as this age group represented a mean proportion of 29.9% (SD 14.3%) of their patients, compared with 15.9% (SD 12.4%) for naturopaths (n = 101) and 7.2% (SD 7.0%) for acupuncturists (n = 84).

The main reasons for consultation, reported by therapists, differed across professions. Low back pain was the most common reason for patients to consult an osteopath (reported by 79.1%of osteopaths, n = 182), followed by neck pain (73.9%, n = 170) and thoracic spine pain (22.2%, n = 51). For acupuncturists, the most common reasons were pain (reported by 45.9% of acupuncturists, n = 39), sleeping disorders (37.6%, n = 32), and musculoskeletal problems (35.3%, n = 30), while for naturopaths the reasons were digestive disorders (reported by 40.6% of naturopaths, n = 41), musculoskeletal problems (34.7%, n = 35), and mood disorders (including stress, anxiety, and depression) (34.7%, n = 35).

### Exploration of practice characteristics

As a secondary objective, we explored factors that influenced therapists’ practices, such as age, gender, and years in practice. Male therapists (n = 136) were in practice for a significantly longer time than female therapists (n = 284) were (15.5 years [SD 8.1] vs. 11.2 [SD 6.7], respectively; p<0.001), but male therapists (n = 132) were also significantly older than female therapists (n = 275) (48.8 years [SD 10.8] vs. 44.6 [SD 11.7], respectively; p<0.001). On average, women worked fewer hours per week and performed fewer consultations per month than men did. However, their consultations lasted longer than those of male therapists (Table 3).

Therapists whose main professional activity was osteopathy, acupuncture, or naturopathy had been in practice longer but were younger than those for whom it was not the main professional activity. Therapists with additional training in another CM, as well as therapists trained in a field other than CM, were significantly older and had been practicing longer than therapists without such trainings. Therapists who were employees were significantly younger and had been in practice for less time than had self-employed therapists. Detailed results are presented in Table 5.

**Table 5:** Practice profile according to gender, age, and years in practice.

Variables		Gender				Age			Years in practice		
		*N*	Male (Mean (SD) or proportion)	Female (Mean (SD) or proportion)	P-value	*N*	Pearson’s correlation or mean (SD)	95% CI or p-value	*N*	Pearson’s correlation or mean (SD)	95% CI or p-value
Weekly working hours		*390*	36.0 (12.8)^a^	27.9 (11.3)^a^	<0.001	*382*	-0.092^c^	-0.19, 0.00^d^	*393*	0.181^c^	0.08, 0.27^d^
Consultations/month		*349*	130.6 (71.4)^a^	79.7 (48.9)^a^	<0.001	*345*	-0.096^c^	-0.20, 0.01^d^	*351*	0.153^c^	0.05, 0.25^d^
Consultation length		*415*	48.9 (13.2)^a^	58.1 (16.1)^a^	<0.001	*402*	0.283^c^	0.19, 0.37^d^	*416*	-0.076^c^	-0.17, 0.02^d^
CM as main professional activity	Yes	*417*	84.4%^b^	81.9%^b^	0.58	*404*	44.7 (11.7)^a^	<0.001^e^	*417*	13.1 (7.6)^a^	0.02^e^
	No		15.6%^b^	18.1%^b^			51.2 (9.8)^a^			10.8 (6.9)^a^	
Employment status	Self-employed	*404*	93.1%^b^	87.9%^b^	0.12	*391*	47.6 (11.1)^a^	<0.001^e^	*405*	13.5 (7.4)^a^	<0.001^e^
	Employee		6.9%^b^	12.1%^b^			33.3 (7.5)^a^			6.5 (6.1)^a^	
Additional CM training	Yes	387	27.4%^b^	35.7%^b^	0.11	375	52.4 (10.4)^a^	<0.001^e^	387	14.2 (8.4)^a^	<0.01^e^
	No		72.6%^b^	64.3%^b^			42.7 (10.4)^a^			11.9 (7.0)^a^	
Former training outside CM	Yes	*410*	61.7%^b^	54.9%^b^	0.20	*401*	51.4 (9.8)^a^	<0.001^e^	*413*	13.5 (7.6)^a^	<0.01^e^
	No		38.3%^b^	45.1%^b^			38.7 (9.8)^a^			11.4 (7.3)^a^	

CI: confidence interval; CM: complementary medicine.

^a^ Results are expressed as mean (SD).

^b^ Results are expressed as percentage of respondents.

^c^ Pearson’s correlation.

^d^ 95% CI.

^e^ p-value.

As there were significant differences in therapists’ profiles, we performed a multivariate analysis, adjusting for profession, age, gender, and interactions between the latter three. Overall, acupuncturists worked significantly fewer hours than osteopaths did, and women worked significantly fewer hours than men did. Acupuncturists and naturopaths performed significantly fewer consults per month than osteopaths did. Women performed fewer consults per month than men did, especially naturopaths and acupuncturists, when interactions between gender and profession were considered. Consultations were significantly longer for acupuncturists and naturopaths than for osteopaths, as well as for female therapists in general. Being a female naturopath involved even longer consults (see Table 6 for detailed results).

**Table 6:** Determinants of workload: Multivariate analysis (linear regressions).

Oucome variable^a^	Explanatory variables	β coefficient	95% CI
Weekly working hours (N = 380)	Intercept	38.25	35.79, 40.71
	Naturopaths	-0.38	-6.40, 5.63
	Acupuncturists	-8.16***	-12.69, -3.64
	Female therapists	-7.03***	-10.23, -3.82
	Age (per year)	-0.04	-0.16, 0.07
	Female therapists x naturopaths Female therapists x acupuncturists	-5.59	-12.42, 1.23
		-2.34	-8.32, 3.64
Consultations/month (N = 343)	Intercept	143.17	131.34, 155.01
	Naturopaths	-41.33**	-68.78, -13.87
	Acupuncturists	-24.84*	-47.53, -2.16
	Female therapist	-37.17***	-52.53, -21.81
	Age (per year)	0.12	-0.43, 0.67
	Female therapists x naturopaths	-27.94	-59.37, 3.49
	Female therapists x acupuncturists	-28.41	-58.33, 1.49
Consultation length (N = 400)	Intercept	42.95	40.72, 45.17
	Naturopaths	18.86***	13.28, 24.43
	Acupuncturists	15.98***	11.92, 20.03
	Female therapists	4.60**	1.72, 7.47
	Age (per year)	-0.17*	-0.33, -0.00
	Female therapist x naturopaths	8.26*	1.82, 14.71
	Female therapist x acupuncturists	0.82	-4.58, 6.23
	Female therapist x age	0.25*	0.05, 0.46

CI: confidence interval; CM: complementary medicine.

^a^ The reference is male osteopaths of 45 years of age.

*** p≤0.001

**p≤0.01

*p≤0.05.

Interpretation example for the number of weekly working hours: the linear regression model for the number of weekly working hours (WWH) is WWH = 38.25−0.38*Natu−8.16*Acu−7.03*Fem−0.04*(Age−45) −5.59*Fem*Natu−2.34*Fem*Acu. The first number (Intercept), 38.25, has to be interpreted as the mean number of weekly working hours for the reference profile. To estimate the mean value of WWH for naturopaths, we consider that the variable “Naturopaths” is equal to 1 and the number −0.38 (regression coefficient or beta weight) has to be added to 38.25. In a similar way, −7.03 is added for female therapist. The coefficient −0.04 associated to the variable Age has to be understood as the increase per year of difference with the reference profile. Thus we will add 0.25 = (−0.05)*(−5) for a 40 year old therapists. For profiles who differ from the reference profile on more than one explanatory variable, it will be necessary to add interaction coefficients (if present). For example, we will add −5.59 in addition to the coefficients −0.38 and −7.03 for female naturopaths. Finally, the estimated mean number of weekly working hours for a 40-year-old female naturopath will be 25.5 = 38.25−0.38−7.03+0.25−5.59

CM was significantly less often the main professional activity for naturopaths and acupuncturists than it was for osteopaths. Osteopaths were significantly more often self-employed than acupuncturists were. Age was positively associated with the likelihood of being a self-employed therapist, especially among osteopaths.

Regarding training, acupuncturists and naturopaths were significantly more likely to have additional training in another CM than osteopaths were. Acupuncturists and naturopaths were more likely than osteopaths to have undertaken previous training outside CM. Age was associated with a greater likelihood of such training for osteopaths, but a smaller likelihood for acupuncturists and naturopaths. Detailed results are presented in Table 7.

**Table 7:** Determinants of working conditions and CM training. Multivariate analysis (logistic regressions).

Outcome variable^a^	Explanatory variables	β coefficient	OR	95% CI
CM as main professional activity (N = 402)	Intercept	2.67		
	Naturopaths	-1.50	0.22***	0.10, 0.47
	Acupuncturists	-1.97	0.14***	0.06, 0.28
	Age (per year)	-0.02	0.97	0.95, 1.00
Self-employment (N = 391)	Intercept	5.14		
	Naturopaths	-0.87	0.42	0.03, 10.76
	Acupuncturists	-2.59	0.08*	0.01, 0.50
	Age (per year)	0.34	1.40***	1.25, 1.62
	Naturopaths’ age (per year)	-0.28	0.76*	0.60, 0.96
	Acupuncturists’ age (per year)	-0.30	0.74***	0.62, 0.87
Additional CM training (N = 374)	Intercept	-2.50		
	Naturopaths	2.79	16.22***	8.12, 33.69
	Acupuncturists	2.26	9.62***	5.01, 19.02
	Female therapists	0.42	1.53	0.83, 2.82
	Age (per year)	0.05	1.05***	1.03, 1.08
Former training outside CM (N = 399)	Intercept	-0.09		
	Naturopaths	1.56	4.76***	2.33, 10.25
	Acupuncturists	1.28	3.58***	1.80, 7.56
	Female therapists	0.13	1.14	0.65, 2.02
	Age (per year)	0.10	1.11***	1.06, 1.16
	Naturopaths’ age (per year)	-0.11	0.90**	0.84, 0.96
	Acupuncturists’ age (per year)	-0.14	0.87***	0.81, 0.94
	Female therapists’ age (per year)	0.09	1.09**	1.03, 1.16

OR: odds ratio; CI: confidence interval; CM: complementary medicine.

^a^The reference is male osteopaths of 45 years of age.

*** p≤0.001

**p≤0.01

*p≤0.05.

Interpretation example for the proportion of therapists who are self-employed: the logistic regression model for the proportion of therapists who are self-employed (SE) is logit(SE) = 5.14−0.87*Natu−2.59*Acu+0.34*(Age−45)−0.28*Natu*(Age−45)−0.30*Acu*(Age−45). In logistic regression, the β weights are not directly interpreted. However, their exponentials (e^β^) can be interpreted as odds ratios. Thus, for a 45-year-old male naturopath, the odds of being self-employed are equal to 0.42 times (58% less) those of a 45-year-old male osteopath (reference profile). For profiles who differ from the reference profile on more than one explanatory variable, one multiplies the corresponding odds ratios. The odds ratio of a 55-year-old acupuncturist would be 0.11 = 0.08*1.40^10^*0.74^10^.

## Discussion

Our results showed that, regardless of their profession, most therapists were women, Swiss, trained in Switzerland, and self-employed. Osteopaths were younger than acupuncturists and naturopaths, worked more weekly hours, performed more consults per month, and had shorter consultations. They were also less likely than acupuncturists or naturopaths to be trained in another CM modality or to have undertaken previous training outside the CM field.

It is difficult to compare our results with those from other countries, as they partly depend on the socio-sanitary context, degree of recognition of these professions, and type of training and diploma available. However, as aspects of these professions are more linked to the professions themselves than to the context in which they occur, we will discuss and compare some of these aspects profession by profession.

### Naturopaths’ professional profile

The results showed a high proportion of female therapists among naturopaths, which is in line with previous studies conducted in Australia, Germany, or the United States [7, 10, 28].

In our sample, a large majority of naturopaths had previous training outside the CM field (85%). In Australia, Bensoussan, Myers (10) found that 31% of naturopaths had training outside naturopathy, 11% of this training pertaining to health. That difference might be explained by the variability in professional recognition in these countries. Indeed, until recently, there was no nationally recognized naturopathic diploma in Switzerland, whereas Australia has offered advanced diplomas and bachelor degrees in naturopathy since the 1990s [13, 29]. Given the situation in Switzerland, we hypothesize that most naturopaths first chose to train in a regulated profession before turning to naturopathy.

Regarding practice characteristics, naturopaths in the present study worked a mean of 24.6 hours a week, which is similar to that reported in other studies [10, 13]. However, the number of consultations per month and the length of consultations differed from previous findings. In our study, naturopaths performed 54 consultations per month, whereas in other countries, this number was higher, at 20 to 27 consultations per week [7, 10, 13, 28]. The length of consultation reported in those studies–ranging from 13.4 min ± 6.3 to 60.3 min ± 22.6—was, however, shorter than that in our population [10, 28, 30].

Regarding the main reasons for patients to consult them, naturopaths in our sample mentioned musculoskeletal problems, mood disorders (including stress, anxiety, and depression), and digestive disorders. These complaints partially matched those found by Cherkin [30] in four American states, who reported fatigue, back symptoms, and anxiety or depression. In Australia, the most common complaints were psychological, gynecological, and endocrine problems [13]. However, the classification of patients’ main complaints may differ from one study to another.

### Acupuncturists’ professional profile

The majority of acupuncturists in our sample were female, which corresponds to findings in other studies [14, 20, 22]. Acupuncturists in our study were often trained in another healthcare-related field (42%). This aspect was also investigated in the US states of Massachusetts and Washington [7], which showed slightly lower rates of other healthcare training (16% of acupuncturists in Massachusetts and 33% in Washington).

No study has indicated the number of weekly working hours, although data are available on the number of consultations. In our sample, acupuncturists performed a mean of 83 consultations per month. Other studies showed rates approaching that, with results ranging from 15 to 25 consultations per week [7, 8, 14, 20]. We found only one study that indicated the mean length of consultations, which was between 41 and 60 minutes [14], similar to our findings.

The main reasons to consult were musculoskeletal problems, pain, and sleeping disorders. Other studies identified broadly similar complaints: musculoskeletal problems [20, 31], pain [22], back and neck symptoms [30], mental health problems [20, 22, 30, 31], fatigue and headaches [30, 31], and obstetrics/gynecology [20, 22]. Previous studies reported that patients used acupuncture for the same general categories of health problems as they did in our study, except for obstetrics/gynecology, which was less often cited in ours.

### Osteopaths’ professional profile

Slightly less than two thirds of osteopath respondents were female. This percentage is higher than findings in previous studies, which indicated rates ranging from 28% to 58% [9, 15, 21, 23, 24]. Contrary to what we observed in our results, in a study performed in Belgium, the Netherlands, and Luxembourg, van Dun et al. [23] found that 85% of osteopaths had previous training as physiotherapists and 11% had another type of previous training. In our study, only about one third of osteopaths had previous training, most often as physiotherapists. The high proportion of former training for osteopaths in the above-mentioned countries might be explained by the fact that there, the profession is not regulated, whereas in Switzerland, osteopathy is regulated at the federal level and recognized as a healthcare profession since 2016 but was already well accepted in the healthcare system before that [24]. Hence, we can hypothesize that physiotherapy—a manual therapy and regulated healthcare profession—might be an entry point for therapists wanting to become osteopaths, as it could provide them with access to official funding models or referral pathways, all the more so in countries without regulation.

In our sample, a large majority of osteopaths were self-employed, which is in line with what van Dun, Nicolaie [23] found in three European countries and with a recent Swiss study [24]. In addition, 71% of osteopaths had one practice location, which is more than that reported in other studies in which 59%-65% of osteopaths had one practice location [9, 15]. Regarding time spent in practice per week, our results (30 h/week) matched those of previous studies that reported between 25 h/week and 33 h/week [15, 21, 23]. However, the mean number of consultations per month was lower in our study (117 consultations per month) compared with that of other studies, with numbers ranging from 33 to 42 consultations per week [9, 15, 21, 23, 24]. These results may indicate more time spent on administrative tasks by the osteopaths in our study. We found only one study that indicated the mean length of consultations. In that study, Morin and Aubin [32] found a mean length of treatment that was similar to that shown in our study.

Back pain, especially low back pain and neck pain, was the most common reason for which patients consulted an osteopath, which is consistent with findings in other countries and confirms a recent Swiss study [9, 11, 24, 32].

### Comparison between professions

Overall, the professional profile of osteopaths differed from the profiles of naturopaths and acupuncturists. These differences were mainly found in previous training, hours worked per week, length of treatments, patients’ complaints, and attitude toward reimbursement of treatments from basic health insurance.

Regarding previous training, we found that acupuncturists and naturopaths were significantly more often trained in an additional CM modality than osteopaths were. This result seems logical in the sense that naturopathy and TCM (which comprises acupuncture) are whole medical systems that include several treatment modalities. Indeed, acupuncturists and naturopaths predominately indicated being trained in CM in consistency with their profile (e.g., naturopaths were often trained in herbal medicine and homeopathy, whereas acupuncturists were often trained in Chinese herbal medicine and Tuina). On the other hand, as osteopathy is a single method, few therapists were trained in another CM modality.

Over two thirds of acupuncturists and naturopaths, but less than one third of osteopaths, had previous training outside the CM field. We hypothesize that this is due to differences in educational status and regulation of these professions. Indeed, in Switzerland, there exists strong heterogeneity among schools and training curricula for acupuncture and naturopathy, and such training has not been regulated until recently. In contrast, osteopathy has been legally recognized as a health profession since 2016 and taught at the university level since 2014; before that, there was only one private osteopathy school in the country that delivered recognized diplomas. Moreover, osteopathic training curricula, unlike acupuncture and naturopathy, is only full-time training. These differences may have an impact on the decision to train in osteopathy directly after high school, whereas becoming a naturopath or an acupuncturist might be a career change, especially for naturopaths, who have less often been trained in another healthcare-related profession. This could also explain why osteopaths were younger than naturopaths and acupuncturists.

Differences were also found in patients’ main complaints, as reported by our participants. Although patients mainly consulted an osteopath for problems related to back pain, those who consulted acupuncturists and naturopaths often reported musculoskeletal problems, as well as mood and sleeping disorders. Thus, it seems that acupuncturists’ and naturopaths’ treatments encompass a wider range of health problems, or at least that their patients visit for a wider range of health problems. Additionally, there is some evidence in the literature regarding the effectiveness these therapies in the treatment of some of the complaints reported by the therapists: osteopathy for the treatment of back pain [33, 34]; acupuncture for the treatment of musculoskeletal problems [35, 36]; and naturopathy for the treatment of mood and sleeping disorders [37, 38].

Finally, therapists’ positions differed regarding reimbursement by basic health insurance. While acupuncturists and naturopaths mainly favored reimbursement of their treatments, osteopaths showed the opposite trend. We can hypothesize that there is a different degree of recognition and regulation of these professions in Switzerland because acupuncture and naturopathy are not regulated at the federal level. Being reimbursed by basic health insurance would thus imply better recognition of these professions. On the other hand, osteopathy is already regulated and recognized as a health profession, making it less essential to be reimbursed through basic health insurance.

### Limitations

Our study has several limitations. First, the response rate was low, thus hindering the possibility to confidently extrapolate the results to the entire population of therapists in the French-speaking part of Switzerland. Second, we investigated only those therapists working in the French-speaking part of Switzerland, as organizational contingencies did not allow to conduct the study over the whole of Switzerland, in two other languages (German and Italian). Third, only a few acupuncturists in our sample were Chinese or trained in China. The fact that our questionnaire was in French may have affected their response rate as a result of language barriers. Thus, Chinese acupuncturists might be underrepresented. Finally, female therapists were overrepresented among the respondents in all three professions (67.8%) compared with our original sample (56.5%).

## Conclusion

This first study to investigate a variety of therapists’ profiles in Switzerland provides useful information about their activities and role within the Swiss healthcare system. Overall, professional profiles differed among therapies. Although all three professions are encompassed under the same umbrella term (CM), our study showed that they have specific work cultures and areas of intervention in the healthcare system. Along with providing greater knowledge about the providers of three widely popular therapies in Switzerland, this study will serve as a basis to further explore particular aspects of those professions (referral patterns, collaboration with other healthcare professions). It would also be useful to better understand which CM characteristics are the same between countries, or differ between countries or culture where CM therapists practice. Along with providing greater knowledge about the providers of three widely popular therapies in Switzerland, this study will serve as a basis to further explore particular aspects of those professions.

## References

[1] Clarke TC, Black LI, Stussman BJ, Barnes PM, Nahin RL. Trends in the use of complementary health approaches among adults: United States, 2002–2012. National health statistics reports. 2015;(79):1–16. Epub 2015/02/12. 25671660 PMC4573565

[2] Steel A, McIntyre E, Harnett J, Foley H, Adams J, Sibbritt D, et al Complementary medicine use in the Australian population: Results of a nationally-representative cross-sectional survey. Sci Rep. 2018;8(1):17325–. 10.1038/s41598-018-35508-y . 30470778 PMC6251890

[3] Eardley S, Bishop FL, Prescott P, Cardini F, Brinkhaus B, Santos-Rey K, et al A systematic literature review of complementary and alternative medicine prevalence in EU. Forschende Komplementarmedizin (2006). 2012;19 Suppl 2:18–28. Epub 2012/01/01. 10.1159/000342708 . 23883941

[4] Klein SD, Torchetti L, Frei-Erb M, Wolf U. Usage of Complementary Medicine in Switzerland: Results of the Swiss Health Survey 2012 and Development Since 2007. PLoS One. 2015;10(10):e0141985 Epub 2015/10/30. 10.1371/journal.pone.0141985 26513370 PMC4626041

[5] von Ammon K, Frei-Erb M, Cardini F, Daig U, Dragan S, Hegyi G, et al Complementary and alternative medicine provision in Europe—first results approaching reality in an unclear field of practices. Forschende Komplementarmedizin (2006). 2012;19 Suppl 2:37–43. Epub 2012/01/01. 10.1159/000343129 . 23883943

[6] Hostettler S, Kraft E. Statistique médicale 2017 de la FMH. Bulletin des médecins suisses. 2018;99(13–14):408–13.

[7] Cherkin DC, Deyo RA, Sherman KJ, Hart LG, Street JH, Hrbek A, et al Characteristics of licensed acupuncturists, chiropractors, massage therapists, and naturopathic physicians. The Journal of the American Board of Family Practice / American Board of Family Practice. 2002;15(5):378–90. Epub 2002/09/28. . 12350060

[8] Floden L, Howerter A, Matthews E, Nichter M, Cunningham JK, Ritenbaugh C, et al Considerations for practice-based research: a cross-sectional survey of chiropractic, acupuncture and massage practices. BMC complementary and alternative medicine. 2015;15:140 Epub 2015/05/03. 10.1186/s12906-015-0659-7 25933801 PMC4429345

[9] Adams J, Sibbritt D, Steel A, Peng W. A workforce survey of Australian osteopathy: analysis of a nationally-representative sample of osteopaths from the Osteopathy Research and Innovation Network (ORION) project. BMC health services research. 2018;18(1):352 Epub 2018/05/12. 10.1186/s12913-018-3158-y 29747647 PMC5946419

[10] Bensoussan A, Myers SP, Wu SM, O'Connor K. Naturopathic and Western herbal medicine practice in Australia-a workforce survey. Complementary therapies in medicine. 2004;12(1):17–27. Epub 2004/05/08. 10.1016/j.ctim.2004.01.001 . 15130568

[11] Burke SR, Myers R, Zhang AL. A profile of osteopathic practice in Australia 2010–2011: a cross sectional survey. BMC Musculoskelet Disord. 2013;14:227 Epub 2013/08/07. 10.1186/1471-2474-14-227 23915239 PMC3733950

[12] Cottingham P, Adams J, Vempati R, Dunn J, Sibbritt D. The characteristics, experiences and perceptions of naturopathic and herbal medicine practitioners: results from a national survey in New Zealand. BMC complementary and alternative medicine. 2015;15:114 Epub 2015/04/19. 10.1186/s12906-015-0616-5 25888473 PMC4405865

[13] Lin V, McCabe P, Bensoussan A, Myers S, Cohen M, Hill S, et al The practice and regulatory requirements of naturopathy and western herbal medicine in Australia. Risk Manag Healthc Policy. 2009;2:21–33. Epub 2009/01/01. 10.2147/RMHP.S4652 22312205 PMC3270908

[14] Moore A, Komesaroff PA, O'Brien K, Xu H, Bensoussan A. Chinese Medicine in Australia. Journal of alternative and complementary medicine (New York, NY). 2016;22(7):515–25. Epub 2016/05/25. 10.1089/acm.2015.0260 . 27219354

[15] Orrock P. Profile of members of the Australian Osteopathic Association: Part 1 –The practitioners. International Journal of Osteopathic Medicine. 2009;12(1):14–24. 10.1016/j.ijosm.2008.04.002

[16] Steel A, Leach M, Wardle J, Sibbritt D, Schloss J, Diezel H, et al The Australian Complementary Medicine Workforce: A Profile of 1,306 Practitioners from the PRACI Study. Journal of alternative and complementary medicine (New York, NY). 2018;24(4):385–94. Epub 2018/01/03. 10.1089/acm.2017.0206 . 29293360

[17] Wardle J, Adams J, Magalhaes RJ, Sibbritt D. Distribution of complementary and alternative medicine (CAM) providers in rural New South Wales, Australia: a step towards explaining high CAM use in rural health? Aust J Rural Health. 2011;19(4):197–204. Epub 2011/07/21. 10.1111/j.1440-1584.2011.01200.x . 21771161

[18] Wardle JL, Barnett R, Adams J. Practice and research in Australian massage therapy: a national workforce survey. International journal of therapeutic massage & bodywork. 2015;8(2):2–11. Epub 2015/06/18. 26082824 10.3822/ijtmb.v8i2.258 PMC4455611

[19] Fawkes CA, Leach CM, Mathias S, Moore AP. A profile of osteopathic care in private practices in the United Kingdom: a national pilot using standardised data collection. Manual therapy. 2014;19(2):125–30. Epub 2013/10/22. 10.1016/j.math.2013.09.001 . 24139392

[20] Hopton AK, Curnoe S, Kanaan M, Macpherson H. Acupuncture in practice: mapping the providers, the patients and the settings in a national cross-sectional survey. BMJ Open. 2012;2(1):e000456 Epub 2012/01/14. 10.1136/bmjopen-2011-000456 22240649 PMC3278493

[21] KPMG. KPMG data survey—How do osteopath practise? 2011.

[22] Robinson N, Lorenc A, Ding W, Jia J, Bovey M, Wang XM. Exploring practice characteristics and research priorities of practitioners of traditional acupuncture in China and the EU-A survey. Journal of ethnopharmacology. 2012;140(3):604–13. Epub 2012/02/18. 10.1016/j.jep.2012.01.052 . 22338645

[23] van Dun PLS, Nicolaie MA, Van Messem A. State of affairs of osteopathy in the Benelux: Benelux Osteosurvey 2013. International Journal of Osteopathic Medicine. 2016;20:3–17. 10.1016/j.ijosm.2016.01.003

[24] Vaucher P, Macdonald RJD, Carnes D. The role of osteopathy in the Swiss primary health care system: a practice review. BMJ Open. 2018;8(8):e023770 Epub 2018/09/03. 10.1136/bmjopen-2018-023770 30173163 PMC6120650

[25] Alvarez Bustins G, Lopez Plaza PV, Carvajal SR. Profile of osteopathic practice in Spain: results from a standardized data collection study. BMC complementary and alternative medicine. 2018;18(1):129 Epub 2018/04/13. 10.1186/s12906-018-2190-0 29642901 PMC5896131

[26] Humphreys BK, Peterson CK, Muehlemann D, Haueter P. Are Swiss chiropractors different than other chiropractors? Results of the job analysis survey 2009. Journal of manipulative and physiological therapeutics. 2010;33(7):519–35. 10.1016/j.jmpt.2010.08.003 20937430

[27] Schwarz I, Hondras MA. A survey of chiropractors practicing in Germany: practice characteristics, professional reading habits, and attitudes and perceptions toward research. Chiropractic & osteopathy. 2007;15:6 Epub 2007/05/08. 10.1186/1746-1340-15-6 17480221 PMC1887523

[28] Goetz K, Kattge S, Steinhäuser J. Satisfied naturopathic practitioners? Results from a job satisfaction survey in the federal state of Schleswig-Holstein, Germany. Eur J Integr Med. 2017;11:41–4. 10.1016/j.eujim.2017.04.001

[29] Baer HA. The drive for legitimation in Australian naturopathy: successes and dilemmas. Social science & medicine (1982). 2006;63(7):1771–83. Epub 2006/06/09. 10.1016/j.socscimed.2006.04.021 . 16759776

[30] Cherkin DC, Deyo RA, Sherman KJ, Hart LG, Street JH, Hrbek A, et al Characteristics of visits to licensed acupuncturists, chiropractors, massage therapists, and naturopathic physicians. The Journal of the American Board of Family Practice / American Board of Family Practice. 2002;15(6):463–72. Epub 2002/12/05. . 12463292

[31] Sherman KJ, Cherkin DC, Eisenberg DM, Erro J, Hrbek A, Deyo RA. The practice of acupuncture: who are the providers and what do they do? Annals of family medicine. 2005;3(2):151–8. Epub 2005/03/31. 10.1370/afm.248 15798042 PMC1466855

[32] Morin C, Aubin A. Primary reasons for osteopathic consultation: a prospective survey in Quebec. PLoS One. 2014;9(9):e106259 Epub 2014/09/04. 10.1371/journal.pone.0106259 25184204 PMC4153609

[33] Chen L, Michalsen A. Management of chronic pain using complementary and integrative medicine. BMJ (Clinical research ed). 2017;357:j1284 Epub 2017/04/26. 10.1136/bmj.j1284 . 28438745

[34] Orrock PJ, Myers SP. Osteopathic intervention in chronic non-specific low back pain: a systematic review. BMC Musculoskelet Disord. 2013;14(1):129 Epub 2013/04/11. 10.1186/1471-2474-14-129 23570655 PMC3623881

[35] Vickers AJ, Linde K. Acupuncture for chronic pain. Jama. 2014;311(9):955–6. 10.1001/jama.2013.285478 24595780 PMC4036643

[36] Lorenc A, Feder G, MacPherson H, Little P, Mercer SW, Sharp D. Scoping review of systematic reviews of complementary medicine for musculoskeletal and mental health conditions. BMJ Open. 2018;8(10):e020222 10.1136/bmjopen-2017-020222 30327397 PMC6196876

[37] Myers SP, Vigar V. The State of the Evidence for Whole-System, Multi-Modality Naturopathic Medicine: A Systematic Scoping Review. The Journal of Alternative and Complementary Medicine. 2019;25(2):141–68. 10.1089/acm.2018.0340 30785315 PMC6389764

[38] Oberg EB, Bradley R, Cooley K, Fritz H, Goldenberg J, Seely D, et al Estimated effects of whole-system naturopathic medicine in select chronic disease conditions: A systematic review. 2015;4(2):1–12.

